# Interstitial duplication of 8q22.1‐q23.1‐ A case report and review of the literature

**DOI:** 10.1002/ccr3.2507

**Published:** 2019-10-24

**Authors:** Steven Leary, Harry S. Porterfield, Jaclyn R. Kotlarek, Benjamin W. Darbro, Alpa Sidhu

**Affiliations:** ^1^ Carver College of Medicine University of Iowa Hospitals and Clinics Iowa City IA USA; ^2^ Department of Pathology University of Iowa Hospitals and Clinics Iowa City IA USA; ^3^ Division of Medical Genetics and Genomics The Stead Family Department of Pediatrics University of Iowa Hospitals and Clinics Iowa City IA USA

**Keywords:** genetics

## Abstract

This report highlights the clinical features seen in duplication of 8q22.1q23.1 inherited from balanced father. It stresses the importance of obtaining a karyotype to identify the location of a large copy number variant for accurate recurrence risk estimation.

## Graphical abstract

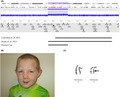

## 1 INTRODUCTION

Here, we present a case of a male patient found to have a derivative chromosome 11 due to an unbalanced translocation with a 12.5 Mb interstitial duplication of 8q22.1q23.1. Comparison of our patient's phenotype to that of reported patients revealed similar facial features and developmental delay.

## 2 CASE REPORT

Interstitial duplications of the long arm of chromosome 8 have rarely been reported in the literature. Among the reported cases, common clinical features include developmental delay, dysmorphic facial features, learning difficulties, congenital heart disease, and feeding issues during infancy^1^, ^2^. In this case report, we present a patient with a 12.5 Mb interstitial duplication of 8q22.1‐q23.1. We compare the reported clinical features with our patient and attempt to identify candidate genes for developmental delay and congenital cardiac defect within the duplicated region.

The proband is a male born full‐term to nonconsanguineous parents with normal birth parameters. The pregnancy was complicated by severe reflux and maternal bipolar disorder for which she was prescribed an antiemetic (ondansetron) and antidepressant (sertraline), respectively. Postnatal course was complicated by inability to latch on breast. He was discharged home in 2 days and was able to take a bottle. Proband was admitted at 1 month of age for failure to thrive and sent home in 2 days. Global developmental delay was noticed within the first year of life with initiation of early intervention therapies. At around 2 years of age, he underwent behavioral and psychological evaluation at our hospital. He did not meet criteria for autism spectrum disorder but was found to have delayed social development and severe speech apraxia requiring the use of a communication device.

**Figure 1:**
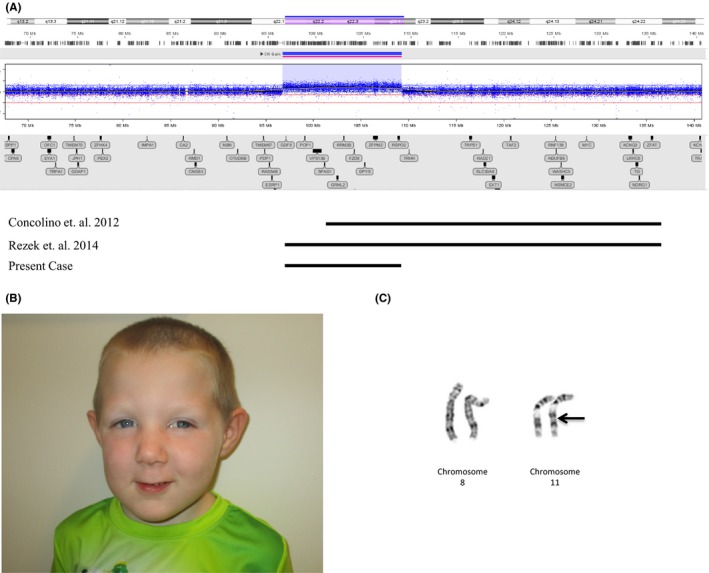
A, Chromosomal microarray depicting the 12.5 Mb duplication of chromosome 8q. Solid lines represent the duplicated region of the present case and compare previously reported cases. B, Facial appearance of our patient at age 4 y. Dysmorphic facial features include down‐slanting palpebral fissures, hypertelorism, hypoplastic eyebrows, broad nasal bridge, open mouth appearance with retrognathia, and a long philtrum. C, Patient chromosome analysis: normal chromosome 8 pair and derivative chromosome 11 showing the interstitial duplication of 8q22.1q23.1 [46, XY, der(11) ins(11;8)(q21;q22.1q23.1)pat]

At a follow‐up developmental visit at three and a half years of age, he was noted to have dysmorphic facial features. Chromosomal microarray (CMA) was sent which demonstrated a pathogenic 12.5 Mb duplication of 8q22.1‐q23.1 (Figure 1A). In addition, there was a 152 kb deletion of 9p21.1 and 55 kb duplication of 15q25.3 (not shown). Neither of these smaller copy number variants (CNVs) had genes linked to human disease and were classified as variants of unknown clinical significance. The family was referred to both cardiology and genetics at that time for further evaluation. An echocardiogram revealed an abnormal right subclavian artery associated with diverticulum of Kommerrell and possible vascular ring. This did not require intervention as the patient remains generally asymptomatic outside of the presence of a murmur.

The patient presented to our genetics clinic at 4 years and 1 month of age to discuss the abnormal chromosomal microarray results. At the time of presentation, he could speak 15‐20 words and used a communication device. He exhibited aggressive and violent behavior with tantrums, lack of impulse control, and tendency for self‐injury. Hearing screens were normal since birth, and he wore corrective glasses for myopia. Family history was significant for post‐traumatic stress and bipolar disorder in mother. On physical exam, the patient was 101.5‐cm tall (37%, *Z* = −0.33 SD), weighed 17.1 kg (63%, *Z* = 0.33 SD), with head circumference of 51 cm (69%, *Z* = 0.50 SD). Facial dysmorphic features included down‐slanting palpebral fissures, hypertelorism, hypoplastic eyebrows, broad nasal bridge, retrognathia, high‐arched palate, and a long philtrum (Figure 1B). The extremities demonstrated thin nails, tapered fingers, normal palmar creases, pes planus, and wide gap between first and second toes.

Targeted parental chromosomal microarray showed that the pathogenic 8q22.1‐q23.1 duplication was not present in either parent. The 55 kb duplication of 15q25.3 was inherited from mother and 152 kb deletion of 9p21.1 from father. Karyotype of the patient resulted in a derivative chromosome 11 with the duplicated 8q22.1‐q23.1 inserted into the long arm of chromosome 11 at band 11q21 [46, XY, der(11) ins(11;8)(q21;q22.1q23.1)] (Figure 1C). Parental karyotypes demonstrated that our patient inherited the duplication from his father who is a balanced carrier of the insertion [46,XY,ins(11;8)(q21;q22.1q23.1)].

## 3 MATERIALS AND METHODS

### 3.1 Karyotype analysis

Lymphocyte cell cultures used in karyotype analysis were obtained from peripheral blood sample of the proband, mother, and father. Cells were arrested in metaphase by adding ethidium bromide (final concentration 12.5 μg/mL) for 40 minutes followed by colcemid (final concentration 6 μg/mL). After 1‐2 hours, the cells were incubated for 25 minutes at room temperature with hypotonic solution (3:1 mixture of 0.8% sodium citrate and 0.075 molar potassium chloride). Cells were then fixed three times with a 3:1 methanol/acetic acid. Twenty G‐banded metaphases were analyzed. Karyotype images were captured on CytoVision computerized imaging system (Applied Imaging).

### 3.2 Chromosomal microarray

Chromosomal oligonucleotide microarray and SNP analysis were done using an Affymetrix CytoScanHD hg19 (NCBI build 37) whole‐genome array consisting of 1.9 million nonpolymorphic markers and 750 000 SNP probes, with an average probe spacing of about 1.2 kb. Data were extracted and processed using Affymetrix ChAS software (Affymetrix; version 1.2.2) and Nexus Copy Number (BioDiscovery; version 7) software.

## 4 DISCUSSION

Interstitial 8q22 duplications are rare with only two reported cases in the literature^1^, ^2^. Here, we report a 4‐year‐old male referred to our clinic with developmental delay, behavioral concerns, and dysmorphic facial features. CMA showed a pathogenic 12.5 Mb duplication of 8q22.1‐q23.1 (Figure 1A). The duplications in the two reported cases are much larger, 49 Mb of 8q22.1‐q24.3 and 44.9 Mb of 8q22.2‐q24.3, making our case the smallest reported so far. DECIPHER database search showed a de novo duplication copy number variant (DECIPHER ID 287285) that partially overlaps our region of interest^3^. This patient is reported in DECIPHER to have conotruncal heart defect, ptosis, and developmental delay. Additional changes seen on our patient's CMA were a 152 kb deletion of 9p21.1 and a 55 kb duplication of 15q25.3. Neither of these regions have any genes linked with human disease and hence, we can say with relative certainty that these would not be contributing to his clinical picture. These copy number variants would be classified as likely benign. Subsequent targeted parental CMA showed that the 12.5 Mb 8q duplication was not present in parents, the 152 kb 9p deletion was paternally, and the 55 kb 15q duplication was maternally, inherited.

Common clinical features of previously reported patients include dysmorphic facial features, intellectual disability, developmental delay, and limb abnormalities. Our patient exhibits dysmorphic facial features of down‐slanting palpebral fissures, hypertelorism, hypoplastic eyebrows, broad nasal bridge, retrognathia, high‐arched palate, and a long philtrum (Figure 1B). Table 1 is a comparison of features seen in our patient with the two other reported interstitial 8q22.1‐q24.3 duplications.

**Table 1:** Clinical manifestations of reported duplication of 8q22‐q24 region in comparison with present case

	Present case	Rezek 2014^2^	Concolino 2012^1^
Duplicated segment	8q22.1‐q23.1	8q22.1‐q24.3	8q22.2‐q24.3
Size	12.5 Mb	49 Mb	44.9 Mb
Location	Derivative chr 11 [46, XY, der(11) ins(11;8)(q21;q22.1q23.1)pat]	Derivative chr 22 [46,XX, der(22) t(8;22)(q22.1;p11.1)mat]	Interstitial duplication
Inheritance	From balanced translocation father	From balanced translocation mother	De novo
Genomic coordinates	hg19 (96779628‐109303690)	hg19 (96871849‐146295771)	hg18 (100338614‐145339830)
Other CMA abnormalities	152 kb del of 9p21.1 (pat) 55 kb dup of 15q25.3 (mat)	NR	−
Facial features			
Hypertelorism	+	+	+
Down‐slanting palpebral fissures	+	NR	−
Hypoplastic eyebrows	+	NR	−
Retrognathia	+	+	+
Cleft lip/palate	−	+	−
Low‐set ears	−	+	+
Long philtrum	+	+	+
Broad nasal bridge	+	+	+
Eye anomalies			
Visual defects	+ (a)	NR	−
Keratoconus, megalocornea	−	NR	+
Birth defects			
Congenital cardiac defect	+ (b)	NR	+ (c)
Cryptorchidism	−	NR	+
Frontal meningocele	−	NR	+
Neurologic anomalies			
Developmental delay	+	+	−
Intellectual delay	+	+	+
Speech apraxia	+	+	+
Seizures	−	+	−
Behavioral abnormalities	+ (d)	NR	−
Growth			
Birth parameters	AGA	NR	AGA
Failure to thrive	+ (e)	NR	+ (f)
Limb anomalies			
Pes planus	+	NR	−
Hypoplastic distal phalanges	+	NR	+
Other	(g)	NR	(h)

Abbreviations: (a), myopia; (b), abnormal right subclavian artery associated with diverticulum of Kommerrell and possible vascular ring; (c), atrial and ventricular septal defects; (d), aggressive and violent, disruptive behavior with lack of impulse control, AGA is appropriate for gestational age; (e), as an infant; (f), <5% at 7 mo; (g), wide gap between first and second toes; (h), syndactyly of second and third toes; −, not present; +, present; NR, not reported.

We next sought to determine the origin of the duplication seen in our patient. Karyotype analysis for the patient showed a derivative chromosome 11 containing the duplicated segment interstitial at band 11q21 (Figure 1C). Parents are interested in having more children and wanted to know accurate recurrence risk for future pregnancy. Parental karyotype showed that our patient inherited the duplication from his father who is a balanced carrier of the insertion. Rezek et al reported the 49 Mb duplication of 8q22.1‐q24.3 in their patient to be inherited from a balanced translocation mother. The derivative chromosome in their patient, however, was acrocentric (chromosome 22), while our patient has an interstitial insertion of the duplication at 11q21. This report highlights the importance of performing a karyotype to identify the location of a large duplicated region and provide accurate recurrence risk to our patients and their families.

We attempted to identify any candidate genes responsible for clinical features seen in our and other reported patients. The duplicated region contains 80 genes with 14 linked to human disorders. ClinGen was used to search for triplosensitivity score for the genes in the duplicated region. None of the genes had a triplosensitivity score above 0. Genes with known function that cause disease in an autosomal recessive manner via loss‐of‐function were excluded from further investigation. We reviewed *RRM2B*, *GDF6*, and *ZFPM2* as potential genes of interest given their known function and association with human disease. The duplicated region of our patient and previously reported two cases include the *RRM2B* and *ZFPM2* genes, while *GDF6* was contained within both our patient and the case described by Rezek et al Duplication of *GDF6* and *SDC2* (not seen in our duplicated segment) has been shown to cause Leri's pleonosteosis characterized by scleroderma‐like congenital rheumatic disease^4^. This would not fit with our patient's clinical picture. Loss‐of‐function mutations in the gene *RRM2B* are known to cause autosomal recessive mitochondrial DNA depletion syndrome and autosomal dominant progressive ophthalmoplegia.^5^, ^6^ There are no reports of a neurodevelopmental phenotype with duplication of this gene. Lastly, loss‐of‐function mutations in *ZFPM2* are associated with Tetralogy of Fallot^7^. There are no reports of duplication of this gene linked with congenital heart disease. Hence, we could not find any conclusive candidate genes explaining the neurodevelopmental phenotype with congenital heart defect, dysmorphic facial features, and limb anomalies seen in our patient.

To our knowledge, this is the third reported case of interstitial 8q22 duplication. Ours is the smallest reported case so far. Interestingly, karyotype analysis showed the duplication to be translocated and inserted into chromosome 11 at an interstitial location, 11q21. Parental studies showed the duplication to be inherited from his father who is a balanced carrier of the insertion. The karyotype result of the proband and parent stresses the importance of performing additional studies to determine location when a large CNV is found on a CMA. This enables the clinical team to give a more accurate recurrence risk estimate to parents if pregnancy is desired in the future. Our case contributes to the known clinical features of this rarely reported duplication and hopes to provide relevant information for genetic counseling and prognosis.

## CONFLICT OF INTEREST

None declared.

## AUTHOR CONTRIBUTION

SL: drafted the original manuscript and table. HSP: searched the Online Mendelian Inheritance in Man (OMIM) database for function and description of genes in the duplicated region. JRK: obtained consent and performed counseling for the testing coordinated in the patient and parents. BWD: contributed to the research testing performed on patient and parental samples. AS: provided patient information, created figure, and performed manuscript revisions.
